# Evaluation of Metal Accumulation in *Escherichia coli* Expressing SPL2 by Single-Cell Inductively Coupled Plasma Mass Spectrometry

**DOI:** 10.3390/ijms26051905

**Published:** 2025-02-22

**Authors:** Yasunori Fukumoto, Enhui Li, Yu-ki Tanaka, Noriyuki Suzuki, Yasumitsu Ogra

**Affiliations:** 1 Graduate School of Pharmaceutical Sciences, Chiba University, Chiba 260-8675, Japannoriyuki.suzuki@phar.toho-u.ac.jp (N.S.); ogra@chiba-u.jp (Y.O.); 2 Faculty of Pharmaceutical Sciences, Toho University, Chiba 274-8510, Japan

**Keywords:** scICP-MS, single-cell inductively coupled plasma mass spectrometry, SPL2, cadmium, bioremediation, biomining

## Abstract

Rare earth elements, comprising 17 elements including 15 lanthanides, are essential components in numerous high-tech applications. While physicochemical methods are commonly employed to remove toxic heavy metals (e.g., cadmium and mercury) from industrial wastewater, biological approaches offer increasingly attractive alternatives. Biomining, which utilizes microorganisms to extract valuable metals from ores and industrial wastes, and bioremediation, which leverages microorganisms to adsorb and transport metal ions into cells via active transport, provide eco-friendly solutions for resource recovery and environmental remediation. In this study, we investigated the potential of three recently identified lanthanide-binding proteins—SPL2, lanpepsy, and lanmodulin—for applications in these areas using single-cell inductively coupled plasma mass spectrometry (scICP-MS). Our results demonstrate that SPL2 exhibits superior characteristics for lanthanide and cadmium bioremediation. Heterologous expression of a cytosolic fragment of SPL2 in bacteria resulted in high expression levels and solubility. Single-cell ICP-MS analysis revealed that these recombinant bacteria accumulated lanthanum, cobalt, nickel, and cadmium, effectively sequestering lanthanum and cadmium from the culture media. Furthermore, SPL2 expression conferred enhanced bacterial tolerance to cadmium exposure. These findings establish SPL2 as a promising candidate for developing recombinant bacterial systems for heavy metal bioremediation and rare earth element biomining.

## 1. Introduction

Rare earth elements (REEs), a group of 17 chemical elements including 15 lanthanides, yttrium, and scandium [1], are indispensable in numerous high-tech applications owing to their unique physicochemical properties [2,3,4]. However, conventional REE mining generates substantial amounts of REE-rich liquid waste, resulting in significant resource loss and posing severe environmental and public health risks [5]. These wastes can contaminate ecosystems and accumulate in the food chain, potentially disrupting normal physiological processes in plants [6] and causing hepatotoxicity and neurotoxicity in humans and animals [7,8,9]. Therefore, developing cost-effective, adaptive, and environmentally sound methods for REE recovery from these liquid wastes is of paramount importance [10].

Biomining, which employs microorganisms to extract valuable metals from ores and mine wastes, has recently emerged as a promising alternative to conventional REE recovery methods. Conventional REE extraction often results in substantial secondary pollution, posing significant environmental challenges [11,12]. In contrast, biomining offers a more sustainable approach. Advances in molecular and genetic engineering have further enhanced the efficiency of REE recovery in biomining processes. A key molecular engineering strategy involves genetically modifying microorganisms to express specific proteins, such as lanthanide-binding tags, which significantly improve REE extraction yields [10,13,14].

Heavy metals, defined as elements with high density and inherent toxicity even at low concentrations [15], pose a significant and growing threat to global environmental and public health [16]. The rapid pace of industrialization and urbanization has dramatically increased the release of these toxic metals into the environment through various anthropogenic activities [17]. While a range of physicochemical processes are currently used for heavy metal removal, these methods are often hampered by significant limitations, including high reagent consumption and the generation of hazardous sludges [18].

Bioremediation, utilizing microorganisms to adsorb and transport metal ions into their cells via active transport [19], offers an eco-friendly alternative to conventional methods for addressing heavy metal pollution. Naturally occurring bacteria, fungi, seaweeds, and algae have demonstrated significant potential for heavy metal adsorption and accumulation [20,21,22,23], offering advantages such as superior adsorption efficiency, the elimination of secondary pollution, and enhanced environmental compatibility compared with physicochemical approaches [24,25]. Microorganisms employ diverse mechanisms for heavy metal detoxification, including cell surface adsorption, intracellular aggregation, mineralization precipitation, and intracellular transformation, even in cases of severe contamination such as with cadmium (Cd) [26,27]. Their small size, ease of culture, and rapid reproduction further contribute to their appeal. However, the practical application of naturally occurring microorganisms can be limited by their often-modest adsorption capacities and stress resistance. To overcome these limitations, recent advances in genetic engineering have focused on enhancing microbial remediation efficiency [28,29]. This involves introducing genes associated with enhanced heavy metal accumulation into robust and genetically tractable recipient strains [30], creating more effective bioremediation agents.

Three proteins with lanthanide-binding potential—SPL2, lanmodulin, and lanpepsy—were investigated in this study. SPL2, a recently identified ubiquitin ligase localized in the outer membrane of plant chloroplasts, has an unclear physiological role. However, its cytosolic fragment exhibits structural similarity to known lanthanide-binding tags and has been shown to bind both lanthanide and calcium (Ca) ions [31]. Lanmodulin, identified in *Methylobacterium extorquens*, is characterized by four metal-binding EF-hand motifs and displays exceptionally high affinity for lanthanides, such as lanthanum (La), terbium (Tb), samarium (Sm), and neodymium (Nd), with dissociation constants in the low picomolar range—eight orders of magnitude lower than those for Ca [32]. Lanpepsy, a small protein induced by La exposure in *Methylobacillus flagellatus*, contains two PepSY domains known for their metal-binding properties. In vitro binding assays suggest that lanpepsy has four lanthanum ion-binding sites and exhibits binding activity for La ions and other lanthanides, with dissociation constants in the low micromolar range [33]. The subcellular localization of lanmodulin and lanpepsy has been predicted to be in the periplasm [32,33]. SPL2, lanpepsy, and lanmodulin were successfully expressed in *Escherichia coli* (*E. coli*) [31,32,33].

Single-cell inductively coupled plasma mass spectrometry (scICP-MS) is a powerful analytical technique that combines the sensitivity of ICP-MS with single-cell resolution necessary for precise quantification of metal concentrations and isotope ratios within individual cells. scICP-MS offers several advantages over bulk analysis using conventional ICP-MS. Namely, acid decomposition is not required, reducing the potential risks of contamination and sample loss. In addition, inaccuracies in cell counting may affect quantification accuracy in a bulk analysis. scICP-MS overcomes these limitations by enabling the direct analysis of individual cells. Moreover, it provides a more comprehensive characterization of samples by showing not only the average elemental content but also the variability and distribution patterns within the population [34,35,36,37,38]. In a recent study, we employed scICP-MS to directly detect histidine-tagged recombinant proteins expressed in bacteria. By labeling these proteins in situ with cobalt (Co) and nickel (Ni) ions, we were able to analyze intact bacterial cells without the need for laborious protein purification steps typically required for in vitro studies. This approach facilitated the characterization of the metal-binding properties of recombinant proteins [39], demonstrating the power of scICP-MS for cellular metalloproteomics.

Of the three lanthanide-binding proteins investigated in this study, SPL2 demonstrated superior characteristics, highlighting its potential for biotechnological applications in both lanthanide biomining and heavy metal bioremediation. Specifically, the metal-binding properties of SPL2 provide a strong scientific foundation for developing recombinant bacterial systems for the effective bioremediation of heavy metal pollution and the sustainable biomining of REEs.

## 2. Results

### 2.1. SPL2 and Lanpepsy Show High Expression and Solubility in Bacteria

**Figure 1:**
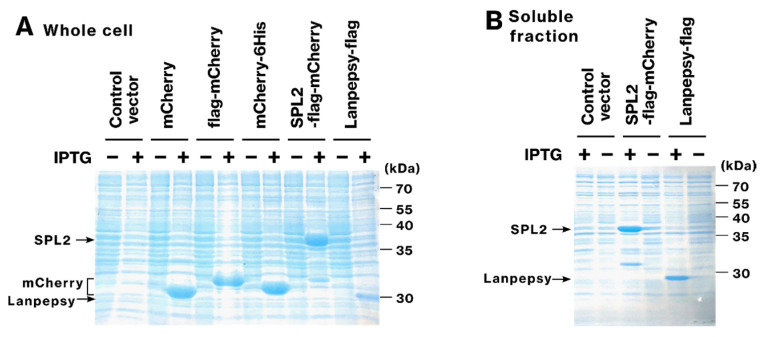
Visualization of recombinant protein expression and solubility in *E. coli*. (**A**) Whole-cell lysates of *E. coli* BL21(DE3) transformed with pET29b plasmids expressing mCherry, flag-mCherry, mCherry-6His, SPL2-flag-mCherry (SPL2 V291–S383), or lanpepsy-flag were analyzed by SDS-PAGE and stained with Coomassie Brilliant Blue (CBB). Recombinant protein expression was induced with IPTG. (**B**) Soluble fractions of the same bacterial lysates, prepared under non-denaturing conditions and clarified by centrifugation, were also analyzed by SDS-PAGE and CBB staining. The calculated molecular weights of flag-mCherry-SPL2 and lanpepsy-flag are 39.7 and 19.3 kDa, respectively.

To examine the expression of potential lanthanoid-binding proteins, we expressed SPL2 (residues V291–S383) fused with flag-mCherry in *E. coli*. Robust expression was observed for both SPL2-flag-mCherry and lanpepsy-flag (Figure 1A). However, bacterial expression of lanmodulin proved challenging; bacteria transformed with the lanmodulin-expression plasmid exhibited significantly impaired growth, and no lanmodulin-flag expression was detected under the tested conditions. Given that our previous work demonstrated that only soluble, and not aggregated, recombinant proteins contribute to bacterial metal-binding ability [39], further analysis focused on soluble proteins SPL2-flag-mCherry and lanpepsy-flag. To confirm the solubility of the recombinant proteins, we analyzed the soluble fraction of bacterial lysates. Sufficient amounts of SPL2-flag-mCherry and lanpepsy-flag were detected (Figure 1B), demonstrating their good solubility and suggesting their potential to function as metal-binding proteins within bacteria.

### 2.2. SPL2 Promotes La Binding in Recombinant Bacteria

**Figure 2:**
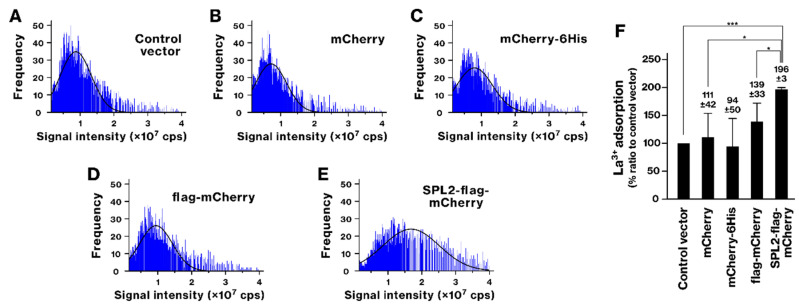
Lanthanum (La) binding in SPL2-expressing bacteria. (**A**–**E**) Recombinant bacteria were cultured in liquid medium, and recombinant protein expression was induced. SPL2-flag-mCherry and control proteins were expressed and then exposed to 250 μM La ion for 1.5 h. Bacterial cells were collected, and La binding was assessed by scICP-MS. The histograms show the frequency distribution of La signal intensities. Representative results from three independent experiments are shown, with Gaussian distribution fitting curves overlaid. (**F**) The amount of La per bacterial cell was calculated from scICP-MS signal intensities, and the average content per cell was determined. The graph shows data from three independent experiments. *, *p* < 0.05. ***, *p* < 0.001.

We employed scICP-MS to investigate the ability of recombinant bacteria to bind lanthanide. Following La exposure, SPL2 expression resulted in a rightward shift in the histogram, accompanied by an increase in the mean and distribution of the signal intensity (Figure 2). The concentration of La ions bound to the bacteria was 28.0 ± 2.7 attomol/cell, whereas in bacteria lacking plasmids, it was 14.3 ± 1.6 attomol/cell. This indicates that the presence of SPL2 led to an approximately twofold increase in La ion binding. In contrast, no such enhancement was observed in bacteria expressing control proteins (mCherry, mCherry-6His, and flag-mCherry). The SPL2-flag-mCherry-expressing group showed a statistically significant increase in La binding compared with control groups. These results demonstrate that the SPL2 fragment facilitates enhanced La binding.

**Figure 3:**
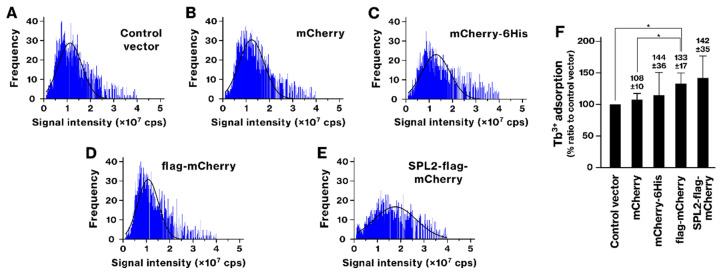
Terbium (Tb) binding by recombinant bacteria. (**A**–**E**) Tb binding analysis by scICP-MS. The experimental procedure was identical to that described in Figure 2, except that the recombinant bacteria were exposed to 250 μM Tb ion. (**F**) The graph shows the average amount of bound Tb per cell. Data represent means from three independent experiments. *, *p* < 0.05.

While SPL2 expression tended to increase Tb binding, this difference was not statistically significant (Figure 3). The flag-mCherry-expressing group also showed increased Tb binding. However, lanpepsy did not enhance the binding of La or Tb (Figure S1).

### 2.3. SPL2 Enhances Transition Metal Binding in Recombinant Bacteria

**Figure 4:**
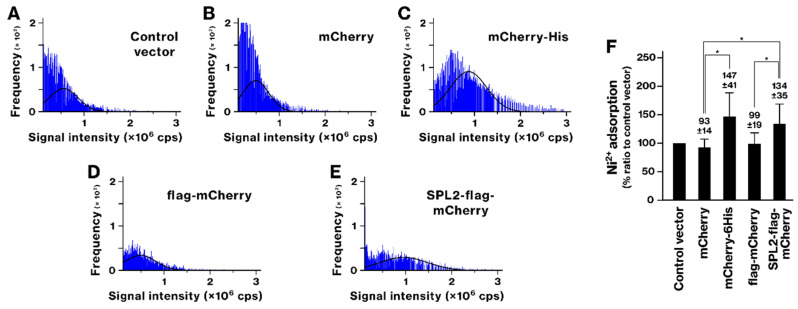
Nickel (Ni) binding by recombinant bacteria expressing mCherry-6His and SPL2-flag-mCherry. (**A**–**E**) The experimental procedure was identical to that described in Figure 2, except that the recombinant bacteria were exposed to 250 μM Ni ion. (**F**) The graph shows the average amount of bound Ni per cell. Data represent means from four independent experiments. *, *p* < 0.05.

**Figure 5:**
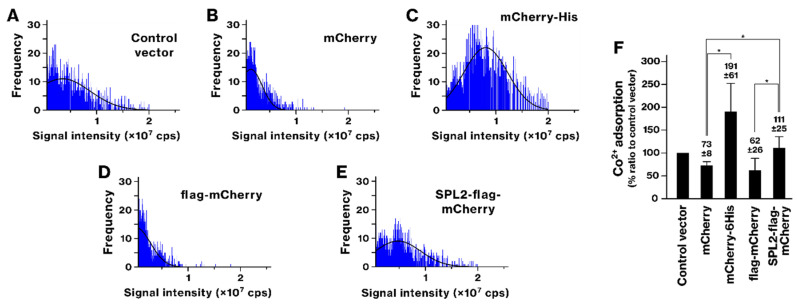
Cobalt (Co) binding by recombinant bacteria expressing mCherry-6His and SPL2-flag-mCherry. (**A**–**E**) The experimental conditions were identical to those described in Figure 2, with the exception that bacteria were exposed to 250 μM Co ion. (**F**) The graph shows the average amount of bound Co per cell. Data represent means from three independent experiments. *, *p* < 0.05.

The ability of SPL2-flag-mCherry-expressing bacteria to bind transition metals was analyzed. Previously, we demonstrated that recombinant bacteria expressing 6His-tagged proteins acquire the ability to accumulate Ni and Co ions [39]. Following exposure to Ni and Co, bacteria expressing mCherry-6His showed a rightward shift in the histogram, whereas those expressing mCherry alone did not (Figure 4 and Figure 5). This indicates that Ni and Co accumulation was mediated by the 6His tags, as shown previously [39]. Ni exposure also caused a rightward shift in the histogram of SPL2-expressing bacteria (Figure 4). The SPL2-flag-mCherry-expressing group exhibited a significant increase in Ni binding compared with the mCherry and flag-mCherry groups, suggesting an association between the SPL2 fragment and Ni. Upon exposure to Co, the histograms of mCherry- or flag-mCherry-expressing bacteria shifted leftward, indicating decreased Co binding (Figure 5). However, SPL2-flag-mCherry expression enhanced Co binding compared with mCherry and flag-mCherry expression. These results suggest a role of the SPL2 fragment in Co binding.

**Figure 6:**
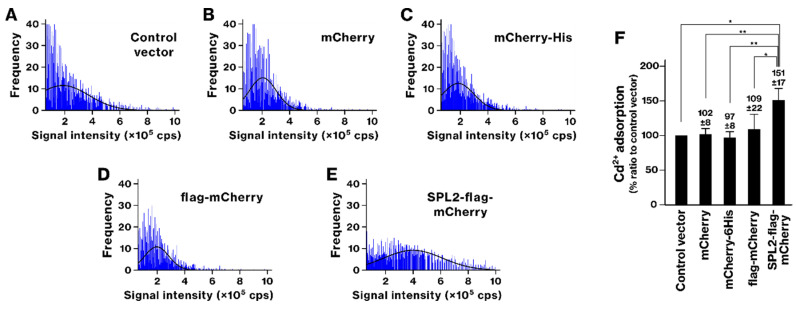
Cadmium (Cd) binding by recombinant bacteria expressing SPL2-flag-mCherry. (**A**–**E**) The experimental procedure was identical to that described in Figure 2, with the exception that the recombinant bacteria were exposed to 250 μM Cd ion. (**F**) The graph shows the average amount of bound Cd per cell. Data represent means from three independent experiments. *, *p* < 0.05. **, *p* < 0.01.

The Cd binding of SPL2-expressing bacteria was also examined. SPL2 expression caused a rightward shift in the histogram (Figure 6). The SPL2-flag-mCherry-expressing group showed a significant increase in Cd binding compared with all other groups. The concentration of Cd ions bound to the bacteria was measured to be 0.15 ± 0.02 attomol/cell. These results indicate that the SPL2 fragment within the SPL2-flag-mCherry protein promoted Cd binding of the recombinant bacteria.

### 2.4. La and Cd Sequestration in SPL2-Expressing Bacteria

**Figure 7:**
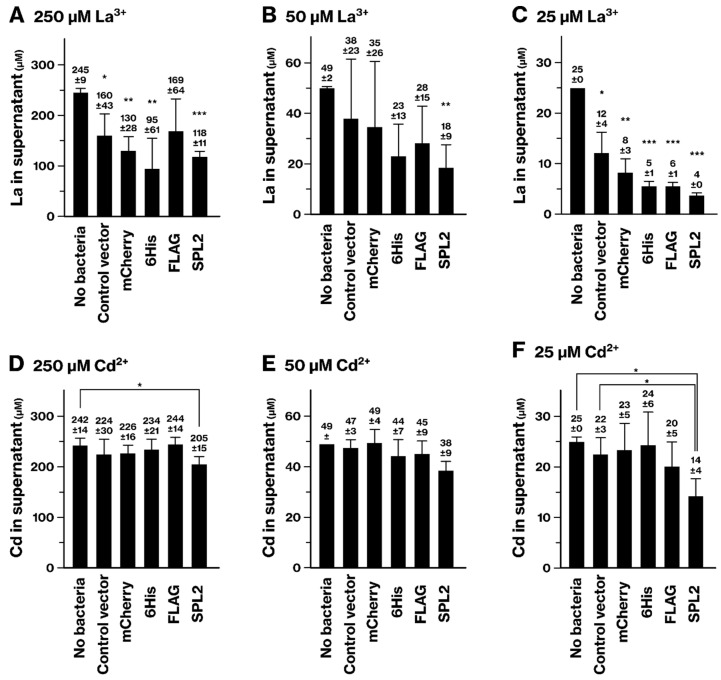
Removal of La and Cd ions from culture media by SPL2-expressing bacteria. (**A**–**C**) La removal. (**D**–**F**) Cd removal. Recombinant bacteria were cultured in liquid medium, and recombinant protein expression was induced. Bacteria were then exposed to the indicated concentrations of La (**A**–**C**) or Cd ions (**D**–**F**) for 1.5 h. Following exposure, the bacterial cells were removed by centrifugation, and metal ion concentrations in the resulting supernatants were measured by ICP-MS. Graphs show means from three independent experiments. *p*-values were calculated using Student’s *t*-test or Welch’s *t*-test, comparing each bacterial group with the no-bacteria control (culture medium without bacteria), except for panel F. 6His, mCherry-6His; FLAG, flag-mCherry; SPL2, SPL2-flag-mCherry. *, *p* < 0.05. **, *p* < 0.01. ***, *p* < 0.001.

We evaluated the ability of recombinant bacteria to remove Cd and La from the culture medium. When cultured in La-containing medium, La concentrations in the culture supernatant generally decreased across all bacterial strains (Figure 7A–C). However, at 250 and 25 μM La, the control vector-containing bacteria showed a significant reduction in La concentration compared with the culture medium not incubated with bacteria, suggesting that La has an affinity for bacterial cells at a baseline level. Remarkably, SPL2-flag-mCherry-expressing bacteria demonstrated a significant reduction in La concentration at all three tested concentrations. This observation indicates that SPL2 expression enhances La sequestration from the culture supernatant beyond the baseline level.

Consistent with the La removal results, Cd concentration in the supernatant showed a significant reduction when the SPL2-expressing bacteria were cultured in media containing 250 μM and 25 μM Cd (Figure 7D–F). The SPL2-expressing group also exhibited a tendency for enhanced Cd removal at 50 μM Cd. These findings suggest that SPL2-expressing bacteria effectively sequester Cd from the culture medium.

### 2.5. SPL2 Expression Increases Cd Tolerance of Recombinant Bacteria

**Figure 8:**
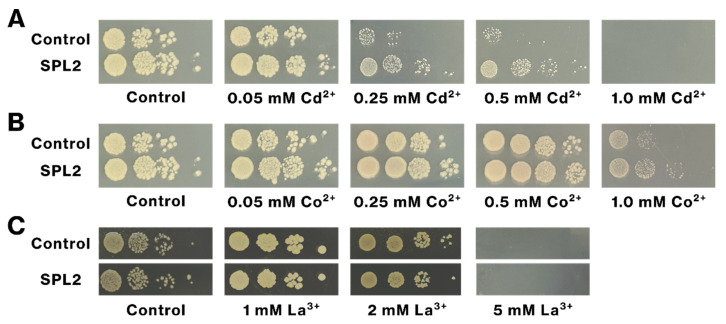
Tolerance of SPL2-expressing bacteria to Cd, Co, and La. Recombinant bacteria expressing SPL2-flag-mCherry or containing the control vector were cultured in liquid medium, and SPL2-FLAG-mCherry expression was induced. Bacteria were serially diluted and spotted on LB agar plates containing the indicated concentrations of Cd (**A**), Co (**B**), or La ions (**C**). Plates were incubated for 12 h, and images were captured. The control vector-containing bacteria served as a negative control. Representative results from more than two independent experiments are shown. Control images for Cd and Co are identical. In panel C, pairs of the control and SPL2 images were taken from the same image.

We examined the impact of SPL2 expression on bacterial tolerance to lanthanides and transition metals. SPL2-expressing bacteria exhibited enhanced growth on Cd-containing plates, compared with control bacteria at Cd concentrations of 0.05 mM, 0.25 mM, and 0.5 mM (Figure 8A). In contrast, SPL2 expression did not promote growth in the presence of Co, La, Tb, or Ni (Figure 8B,C). These results strongly suggest that SPL2 expression specifically confers tolerance to Cd, but not to the other tested metals, in the recombinant bacteria.

## 3. Discussion

Microorganisms are attractive candidates for bioremediation and biomining owing to their efficient and selective metal accumulation. Genetic engineering further enhances their metal-binding ability and selectivity [25,26,27,28,29,30]. In this regard, the expression and solubility of SPL2- and lanpepsy-derived recombinant proteins suggest their potential for genetic engineering. In this study, we expressed three lanthanide-binding proteins—SPL2, lanmodulin, and lanpepsy—in *E. coli* strains to develop metal-accumulating recombinant bacteria. However, lanmodulin expression inhibited bacterial growth, preventing evaluation of its bioremediation potential. As previously shown, recombinant protein solubility is crucial for metal binding [39]. Efficient expression and solubility of SPL2 and lanpepsy in bacterial cells (Figure 1A,B) suggest their suitability for genetic engineering for bioremediation and biomining.

scICP-MS analyses identified SPL2 as a promising candidate for bioremediation and biomining. This technique provides precise detection and quantification of metal ions at the single-cell level, revealing detailed information on their distribution and concentration within individual cells [34,35]. SPL2-expressing bacteria exhibited enhanced binding of several metals, including La, Cd, Co, and Ni (Figure 2, Figure 4, Figure 5 and Figure 6). This enhanced binding was directly attributed to the SPL2 fragment, as bacteria expressing SPL2-flag-mCherry showed a significant increase in metal binding compared with those expressing flag-mCherry alone. Importantly, the enhanced binding translated into effective sequestration of La and Cd ions from the culture supernatant by SPL2-expressing bacteria (Figure 7). These findings highlight the potential of the SPL2 cytosolic fragment (V291–S383) for accumulating metal ions, particularly La and Cd, in recombinant bacteria, suggesting its suitability for bioremediation and biomining applications targeting heavy metals and REE.

We identify SPL2 as a novel candidate for Cd bioremediation, offering an approach distinct from previously reported methods. While one study has described microbial-mediated mineralization of Cd, converting it to insoluble forms, such as carbonate, phosphate salts, and cadmium sulfide [26], and another study has demonstrated Cd adsorption using recombinant bacteria expressing human ferritin fused with a synthetic phytochelatin gene [30], our findings introduce the soluble, cytosolic fragment of SPL2 as a potential tool for Cd adsorption. The presence of SPL2-flag-mCherry in the soluble fraction after cell lysis (Figure 1B) suggests intracellular sequestration and accumulation of Cd. Furthermore, SPL2-flag-mCherry expression enhanced bacterial growth upon Cd exposure (Figure 8), indicating that SPL2 can also improve bacterial resistance to Cd toxicity, further highlighting its potential for Cd bioremediation.

The imidazole group in histidine has been extensively studied for its applications in the removal of toxic metal ions from contaminated water using polymer-based membranes, including potential industrial applications [40]. In our previous study, we showed that recombinant bacteria expressing 6His-tagged proteins developed the capability to accumulate Ni and Co ions [39]. Conversely, while His-tagged proteins proved effective in the bacterial system, the cytosolic fragment of SPL2 may, in turn, be applicable in bioorganic membranes and polymer-based systems.

## 4. Materials and Methods

### 4.1. Plasmid Vectors

To investigate the properties of several proteins, synthetic open reading frames (ORFs) were designed and cloned into the pET29b expression vector. The ORFs were synthesized by Twist Biosciences (San Francisco, CA, USA). The constructs included mCherry without any tags, as well as mCherry fused to a C-terminal hexahistidine (6His) tag (mCherry-6His) or an N-terminal FLAG epitope tag (FLAG-mCherry). Additionally, a fusion protein was generated by attaching a cytoplasmic fragment of SPL2 (V291–S383) to the C-terminus of FLAG-mCherry, resulting in the SPL2-FLAG-mCherry construct. Two additional constructs were designed to express FLAG-tagged versions of lanpepsy (D24–E175) and lanmodulin, referred to as lanpepsy-FLAG and lanmodulin-FLAG, respectively. In the case of lanpepsy, the N-terminal intrinsically disordered region was excluded to generate lanpepsy-FLAG. These constructs were then transformed into *E. coli* BL21(DE3) via electroporation. Transformed bacteria were subsequently cultured on LB agar plates containing 50 μg/mL kanamycin for selection.

### 4.2. Expression and Solubility of Recombinant Proteins

We used the IPTG induction system because it is widely utilized in the field of molecular biology. Recombinant protein expression was performed in *E. coli* BL21(DE) as previously described [41,42]. Briefly, a single bacterial colony was grown overnight at 37 °C in LB medium supplemented with 50 μg/mL kanamycin. The preculture was then diluted and grown at 37 °C until an OD_600_ of 0.4–0.6 was reached. Protein expression was induced with 0.5 mM IPTG (Takara Bio Inc., Shiga, Japan) for 3 h at 37 °C. Cells were harvested by centrifugation and washed once with buffer [50 mM Tris–HCl (pH 7.5), 10% sucrose]. Two distinct lysis methods were employed: for evaluating total protein expression, cells were directly lysed in Laemmli SDS lysis buffer by boiling at 95 °C for 5 min. For assessing protein solubility, cells were suspended in lysis buffer [20 mM NaPi (pH 6.8), 300 mM NaCl, 0.5 mM PMSF, protease inhibitor cocktail], treated with 1 mg/mL lysozyme for 15 min on ice, flash-frozen in liquid nitrogen, and subjected to three freezing-thawing cycles followed by sonication (Tomy, Tokyo, Japan, UR-20P sonicator, level 5, 10 s pulses repeated 10 times on ice). The resulting lysate was clarified by centrifugation, and the supernatant was analyzed by SDS-PAGE and Coomassie Brilliant Blue (CBB) staining.

### 4.3. E. coli Preparation for scICP-MS Analysis

A single colony of the recombinant bacteria was cultured overnight in LB medium supplemented with kanamycin. The culture was grown at 37 °C to mid-logarithmic phase after dilution with fresh medium. Protein expression was then induced with 0.1 mM IPTG, and bacterial cells were cultured at 18 °C for 18 h. To investigate metal binding, bacterial cells were exposed to 250 μM of either lanthanum chloride (FUJIFILM Wako Pure Chemical Corporation, Inc., Osaka, Japan, Cat. No. 123-04222), terbium chloride (FUJIFILM Wako Pure Chemical Corporation, Inc., Cat. No. 206-14491), cadmium chloride (FUJIFILM Wako Pure Chemical Corporation, Inc., Cat. No. 032-00122), nickel chloride (Nacalai Tesque, Inc., Kyoto, Japan, Cat. No. 24223-92), or cobalt chloride (Nacalai Tesque, Inc., Kyoto, Japan, Cat. No. 09208-72) for 1.5 h. To remove unbound metal ions, the exposed cells were washed extensively (three times) with 0.9% sodium chloride solution (99.999%, Sigma-Aldrich, St. Louis, MO, USA, Cat. No. 31319-45) before analysis by scICP-MS.

### 4.4. Metal Analysis of Recombinant Bacteria by scICP-MS

Single-cell ICP-MS analysis was performed as described previously [36,37,39]. Metal ion-exposed bacteria were suspended and introduced into an Agilent 8900 ICP-MS/MS system (Agilent Technologies, Hachioji, Japan) using a dedicated single-cell sample introduction system (Glass Expansion, Melbourne, Australia) consisting of a MicroMist concentric glass nebulizer, a total consumption spray chamber, and a micro syringe pump (MSP-1D, AS ONE, Osaka, Japan). Time-resolved analysis was used to sequentially acquire signals of all elements of interest. The mass (*m*, in grams) of La, Tb, Cd, Co, and Ni in individual bacterial cells was then calculated by comparing the measured signal intensities from the cells (*I*_Cell_) to those from an ionic standard solution (*I*_Std_) using the following equation.

$$m=\frac{I_{Cell}}{I_{Std}-I_{Blk}}\times t_{dwell}\times f\times C_{Std}\times v$$

*I*_Blk_ represents the signal intensity of 0.9% sodium chloride blank solution; *t*_dwell_, the signal integration or dwell time, was set to 0.1 ms; *f*, the nebulization or transport efficiency, was determined using SiO_2_ nanoparticles (200 nm, Sigma-Aldrich, Cat No. 231-545-4) and an ionic silicon standard (Kanto Chemical Co., Inc., Tokyo, Japan, Cat. No. 37811-2B), following the procedure described in our previous study [39]; *C*_Std_ represents the concentration of the ionic standard, set at 100 ng/mL; and *v* represents the sample flow rate, set at 0.015 mL/min. All calculations, including automatic particle baseline calibration, were performed using ICP-MS MassHunter software 5.2 (Agilent Technologies). Detailed operational settings and instrumental conditions for scICP-MS analysis are summarized in Table 1.

In the collision/reaction cell, hydrogen (H_2_) gas was used to analyze La, Tb, Cd, Ni, Co, and Si, while oxygen (O_2_) gas was used to analyze P. We selected the reaction gases for Si, P, Ni, and Co based on our previous manuscript [39]. To minimize spectral interferences arising from atmospheric and solvent-derived contaminants, H_2_ was used as a reaction gas in the analysis of La, Tb, and Cd. This approach is expected to reduce interference from polyatomic species, such as nitrogen- and oxygen-containing ions.

Single-cell data were analyzed using GraphPad Prism 9.0. The mean mass of metal ions in each bacterial cell was determined by fitting a Gaussian distribution to the corresponding histograms. To account for variations between experiments, the mean of each sample was normalized to the mean of the control vector. Data from more than three independent experiments were presented as means ± standard deviation. Statistical significance was assessed using Student’s *t*-test or Welch’s *t*-test, as appropriate. Unless otherwise stated, comparisons were made between the control vector and the mCherry-expressing groups.

### 4.5. Heavy Metal Tolerance of SPL2-Expressing Bacteria

To assess metal tolerance, recombinant bacteria expressing SPL2-flag-mCherry were cultured overnight in LB medium containing 50 μg/mL kanamycin. Bacteria transformed with the empty pET29b vector served as a control. Following dilution of the preculture and growth at 37 °C to the mid-logarithmic phase, SPL2 expression was induced with 0.5 mM IPTG. After a 4 h cultivation, bacterial cultures were serially diluted (2-, 3-, 4-, 5, and 10-fold) with fresh LB medium. Three microliters of each dilution was then spotted onto LB agar plates supplemented with various concentrations of different metal ions. After 12 h at 37 °C, the plates were photographed to assess bacterial growth. A metal-free LB plate served as a negative control.

### 4.6. Removal of Heavy Metals by SPL2-Expressing Bacteria

To analyze metal concentrations in the bacterial growth medium, SPL2-expressing bacteria were cultivated overnight at 37 °C in LB medium supplemented with kanamycin. Following dilution with fresh LB medium and growth to the mid-logarithmic phase, SPL2 expression was induced with 0.1 mM IPTG, and the bacteria were cultured at 18 °C for 18 h. Subsequently, metal ions were added, and the cultures were incubated at 37 °C for 1.5 h. Bacterial cells were then removed by centrifugation (10,000× *g*, 10 min, 4 °C), and the resulting supernatant was collected for metal concentration analysis by ICP-MS. Data from three independent experiments were presented as means ± standard deviation. Statistical significance was determined using Student’s *t*-test or Welch’s *t*-test.

## 5. Conclusions

Our scICP-MS analyses demonstrated that the expression of the cytosolic fragment of SPL2 enhanced the accumulation of several metals—La, Co, and Ni—within bacterial cells. Remarkably, SPL2 also exhibited Cd-binding ability, enabling SPL2-expressing recombinant bacteria to effectively sequester this metal from the culture medium, along with La. Furthermore, SPL2 expression conferred increased tolerance to Cd toxicity exposure in the recombinant bacteria. These multifaceted findings suggest that the metal-binding ability of SPL2 offers a novel and promising strategy for bioremediation and biomining using recombinant bacteria.

## Figures and Tables

**Table 1:** ICP-MS Instrumentation and Collision/Reaction Operational Settings.

Instrument	Agilent 8900 ICP-MS/MS
ICP incident power	1600 W
Ar carrier gas	0.60 L/min
Make-up gas	0.25 L/min
Integration time	0.1 ms
Collision/reaction cell	H_2_: 5.5 mL/min (La, Tb, Cd, Ni, Co, Si)
	O_2_: 0.38 mL/min (P)
Signal monitoring period	40 s
Sample injection rate	0.015 mL/min

## Data Availability

Data is contained within the article or Supplementary Material.

## Supplementary Materials

The following supporting information can be downloaded at: https://www.mdpi.com/article/10.3390/ijms26051905/s1.

## References

[1] Kowalkowski T., Pastuszak M., Szparaga A., Samczynski Z., Polkowska-Motrenko H., Buszewski B. (2019). Rare earth elements in fine fraction (<20 mum) of the Vistula River sediments. Chemosphere.

[2] Zepf V. (2013). Rare Earth Elements: A New Approach to the Nexus of Supply, Demand and Use: Exemplified along the Use of Neodymium in Permanent Magnets.

[3] Dushyantha N., Batapola N., Ilankoon I.M.S.K., Rohitha S., Premasiri R., Abeysinghe B., Ratnayake N., Dissanayake K. (2020). The story of rare earth elements (REEs): Occurrences, global distribution, genesis, geology, mineralogy and global production. Ore Geol. Rev..

[4] Balaram V. (2019). Rare earth elements: A review of applications, occurrence, exploration, analysis, recycling, and environmental impact. Geosci. Front..

[5] Gwenzi W., Mangori L., Danha C., Chaukura N., Dunjana N., Sanganyado E. (2018). Sources, behaviour, and environmental and human health risks of high-technology rare earth elements as emerging contaminants. Sci. Total Environ..

[6] Wang Z., Zhang X., Mu Y. (2008). Effects of rare-earth fertilizers on the emission of nitrous oxide from agricultural soils in China. Atmos. Environ..

[7] Basu A., Chakrabarty K., Chatterjee G.C. (1982). Neurotoxicity of lanthanum chloride in newborn chicks. Toxicol. Lett..

[8] Palasz A., Czekaj P. (2000). Toxicological and cytophysiological aspects of lanthanides action. Acta Biochim. Pol..

[9] Feng L., Xiao H., He X., Li Z., Li F., Liu N., Zhao Y., Huang Y., Zhang Z., Chai Z. (2006). Neurotoxicological consequence of long-term exposure to lanthanum. Toxicol. Lett..

[10] Xie X., Tan X., Yu Y., Li Y., Wang P., Liang Y., Yan Y. (2022). Effectively auto-regulated adsorption and recovery of rare earth elements via an engineered *E. coli*. J. Hazard. Mater..

[11] Abashina T., Vainshtein M. (2023). Current Trends in Metal Biomining with a Focus on Genomics Aspects and Attention to Arsenopyrite Leaching-A Review. Microorganisms.

[12] Liapun V., Motola M. (2023). Current overview and future perspective in fungal biorecovery of metals from secondary sources. J. Environ. Manag..

[13] Park D.M., Reed D.W., Yung M.C., Eslamimanesh A., Lencka M.M., Anderko A., Fujita Y., Riman R.E., Navrotsky A., Jiao Y. (2016). Bioadsorption of Rare Earth Elements through Cell Surface Display of Lanthanide Binding Tags. Environ. Sci. Technol..

[14] Park D.M., Brewer A., Reed D.W., Lammers L.N., Jiao Y. (2017). Recovery of Rare Earth Elements from Low-Grade Feedstock Leachates Using Engineered Bacteria. Environ. Sci. Technol..

[15] Martin K., Hosam M.S., Hosam El-Din M.S., Refaat F.A. (2018). Introductory Chapter: Introducing Heavy Metals. Heavy Metals.

[16] Vhahangwele M., Khathutshelo L.M., Hosam El-Din M.S., Refaat F.A. (2018). Environmental Contamination by Heavy Metals. Heavy Metals.

[17] Armah F.A., Obiri S., Yawson D.O., Onumah E.E., Yengoh G.T., Afrifa EK A., Odoi J.O. (2010). Anthropogenic sources and environmentally relevant concentrations of heavy metals in surface water of a mining district in Ghana: A multivariate statistical approach. J. Environ. Sci. Health A.

[18] Razzak S.A., Faruque M.O., Alsheikh Z., Alsheikhmohamad L., Alkuroud D., Alfayez A., Hossain SM Z., Hossain M.M. (2022). A comprehensive review on conventional and biological-driven heavy metals removal from industrial wastewater. Environ. Adv..

[19] Ramya S.L., Vinay Kumar C., Sudhamani M., Jan D., Branislav V. (2018). Application of Biosorption for Removal of Heavy Metals from Wastewater. Biosorption.

[20] Liu S.H., Zeng G.M., Niu Q.Y., Liu Y., Zhou L., Jiang L.H., Tan X.F., Xu P., Zhang C., Cheng M. (2017). Bioremediation mechanisms of combined pollution of PAHs and heavy metals by bacteria and fungi: A mini review. Bioresour. Technol..

[21] Znad H., Awual M.R., Martini S. (2022). The Utilization of Algae and Seaweed Biomass for Bioremediation of Heavy Metal-Contaminated Wastewater. Molecules.

[22] Salama E.S., Roh H.S., Dev S., Khan M.A., Abou-Shanab RA I., Chang S.W., Jeon B.H. (2019). Algae as a green technology for heavy metals removal from various wastewater. World J. Microbiol. Biotechnol..

[23] Arumugam N., Chelliapan S., Kamyab H., Thirugnana S., Othman N., Nasri N.S. (2018). Treatment of Wastewater Using Seaweed: A Review. Int. J. Environ. Res. Public Health.

[24] Alabssawy A.N., Hashem A.H. (2024). Bioremediation of hazardous heavy metals by marine microorganisms: A recent review. Arch. Microbiol..

[25] Roy R., Samanta S., Pandit S., Naaz T., Banerjee S., Rawat J.M., Chaubey K.K., Saha R.P. (2024). An Overview of Bacteria-Mediated Heavy Metal Bioremediation Strategies. Appl. Biochem. Biotechnol..

[26] Zheng Y., Xiao C., Chi R. (2021). Remediation of soil cadmium pollution by biomineralization using microbial-induced precipitation: A review. World J. Microbiol. Biotechnol..

[27] Anand S., Singh A., Kumar V. (2023). Recent advancements in cadmium-microbe interactive relations and their application for environmental remediation: A mechanistic overview. Environ. Sci. Pollut. Res. Int..

[28] Hansda A., Kumar V., Anshumali (2016). A comparative review towards potential of microbial cells for heavy metal removal with emphasis on biosorption and bioaccumulation. World J. Microbiol. Biotechnol..

[29] Saravanan A., Kumar P.S., Ramesh B., Srinivasan S. (2022). Removal of toxic heavy metals using genetically engineered microbes: Molecular tools, risk assessment and management strategies. Chemosphere.

[30] Tian L., Wang D., Liu Y., Wei M., Han X., Sun X., Yin L., Luo G. (2024). Construction of Genetically Engineered *Escherichia coli* Cell Factory for Enhanced Cadmium Bioaccumulation in Wastewater. Water.

[31] Tracz M., Gorniak I., Szczepaniak A., Bialek W. (2021). E3 Ubiquitin Ligase SPL2 Is a Lanthanide-Binding Protein. Int. J. Mol. Sci..

[32] Cotruvo J.A., Jr Featherston E.R., Mattocks J.A., Ho J.V., Laremore T.N. (2018). Lanmodulin: A Highly Selective Lanthanide-Binding Protein from a Lanthanide-Utilizing Bacterium. J. Am. Chem. Soc..

[33] Hemmann J.L., Keller P., Hemmerle L., Vonderach T., Ochsner A.M., Bortfeld-Miller M., Gunther D., Vorholt J.A. (2023). Lanpepsy is a novel lanthanide-binding protein involved in the lanthanide response of the obligate methylotroph Methylobacillus flagellatus. J. Biol. Chem..

[34] Meyer S., Lopez-Serrano A., Mitze H., Jakubowski N., Schwerdtle T. (2018). Single-cell analysis by ICP-MS/MS as a fast tool for cellular bioavailability studies of arsenite. Metallomics.

[35] Ho K.-S., Chan W.-T. (2010). Time-resolved ICP-MS measurement for single-cell analysis and on-line cytometry. J. Anal. At. Spectrom..

[36] Tanaka Y., Iida R., Takada S., Kubota T., Yamanaka M., Sugiyama N., Abdelnour Y., Ogra Y. (2020). Quantitative Elemental Analysis of a Single Cell by Using Inductively Coupled Plasma-Mass Spectrometry in Fast Time-Resolved Analysis Mode. Chembiochem.

[37] Tanaka Y., Katayama H., Iida R., Ogra Y. (2025). Quantitative elemental analysis of human leukemia K562 single cells by inductively coupled plasma mass spectrometry in combination with a microdroplet generator. J. Anal. At. Spectrom..

[38] Ogra Y., Tanaka Y., Suzuki N. (2022). Recent advances in copper analyses by inorganic mass spectrometry. J. Clin. Biochem. Nutr..

[39] Tanaka Y., Shimazaki S., Fukumoto Y., Ogra Y. (2022). Detection of Histidine-Tagged Protein in *Escherichia coli* by Single-Cell Inductively Coupled Plasma-Mass Spectrometry. Anal. Chem..

[40] Kaczorowska M.A. (2022). The Use of Polymer Inclusion Membranes for the Removal of Metal Ions from Aqueous Solutions-The Latest Achievements and Potential Industrial Applications: A Review. Membranes.

[41] Fukumoto Y., Kyono R., Shibukawa Y., Tanaka Y., Suzuki N., Ogra Y. (2024). Differential molecular mechanisms of substrate recognition by selenium methyltransferases, INMT and TPMT, in selenium detoxification and excretion. J. Biol. Chem..

[42] Fukumoto Y., Nakayama Y., Yamaguchi N. (2019). Human Rad17 C-terminal tail is phosphorylated by concerted action of CK1delta/epsilon and CK2 to promote interaction with the 9-1-1 complex. Biochem. Biophys. Res. Commun..

