# Supplementary material for: Evaluation of Metal Accumulation in Escherichia coli Expressing SPL2 by Single-Cell Inductively Coupled Plasma Mass Spectrometry
Source: Int J Mol Sci. 2025 Feb 22;26(5):1905. doi: 10.3390/ijms26051905 (PMC11899968; doi:10.3390/ijms26051905)
Supplement: Supplementary file 1 [file ijms-26-01905-s001.zip › ijms-3424785-supplementary.pdf]

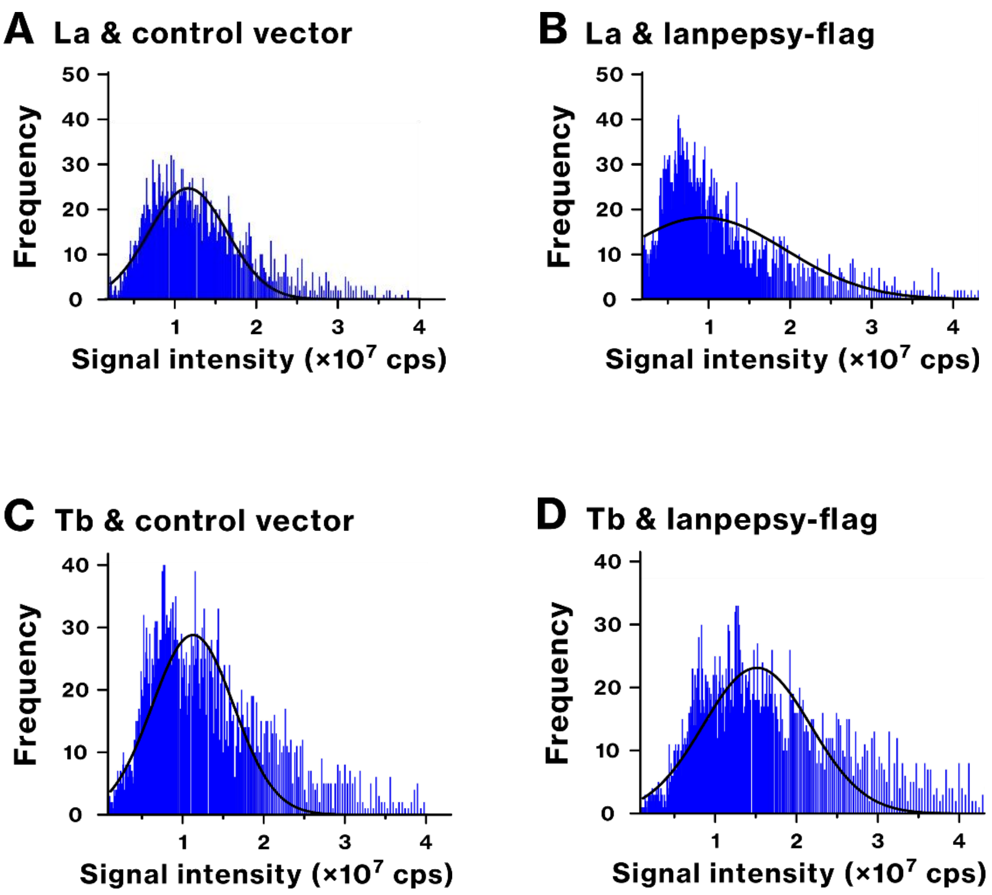

Figure S1. Lanpepsy and binding of La and Tb.

The same experiment as in Figures 2 and 3. Recombinant bacteria were cultured in liquid medium, and recombinant protein expression was induced. Lanpepsy-flag was expressed and then exposed to 250 μM La (A, B) or Tb (C, D) ions for 1.5 hours. Bacterial cells were collected, and metal binding was assessed by scICP-MS. The histograms show the frequency distribution of signal intensities. Representative results from more than two independent experiments are shown, with Gaussian distribution fitting curves overlaid.
